# The Effect of PAMAM Dendrimers on the Antibacterial Activity of Antibiotics with Different Water Solubility

**DOI:** 10.3390/molecules18078607

**Published:** 2013-07-22

**Authors:** Katarzyna Winnicka, Magdalena Wroblewska, Piotr Wieczorek, Pawel Tomasz Sacha, Elzbieta Anna Tryniszewska

**Affiliations:** 1 Department of Pharmaceutical Technology, Faculty of Pharmacy, Medical University of Białystok, Mickiewicza 2c, 15-222 Białystok, Poland; E-Mail: magdalena.wroblewska@umb.edu.pl; 2 Department of Microbiological Diagnostics and Infectious Immunology, Faculty of Pharmacy, Medical University of Białystok, Kilińskiego 1, 15-089 Białystok, Poland; E-Mails: piowie@umb.edu.pl (P.W.); sachpt@umb.edu.pl (P.T.S.); zdmik@umb.edu.pl (E.A.T.)

**Keywords:** PAMAM dendrimer, tobramycin, erythromycin, antibacterial activity, water solubility

## Abstract

Erythromycin (EM) and tobramycin (TOB) are well-known and widely used antibiotics, belonging to different therapeutic groups: macrolide and aminoglycoside, respectively. Moreover, they possess different solubility: EM is slightly soluble and TOB is freely soluble in water. It was previously demonstrated that PAMAM dendrimers enhanced the pharmacological activity of antifungal drugs by increasing their solubility. Therefore, it appears interesting to investigate the effect of PAMAM-NH_2_ and PAMAM-OH dendrimers generation 2 (G2) and generation 3 (G3) on the antibacterial activity of antibiotics with different water solubility. In this study it was shown that the aqueous solubility of EM was significantly increased by PAMAM dendrimers (PAMAM-NH_2_ and PAMAM-OH caused about 8- and 7- fold solubility increases, respectively). However, it was indicated that despite the increase in the solubility, there was only slight influence on the antibacterial activity of EM (2- and 4- fold decreases in the MBC values of EM in the presence of PAMAM-OH G3 and PAMAM-NH_2_ G2 or G3 for strains of *Staphylococcus aureus* were noted, respectively). It was also found that there was no influence of PAMAM on the antibacterial activity of hydrophilic TOB.

## 1. Introduction

**Figure 1:**
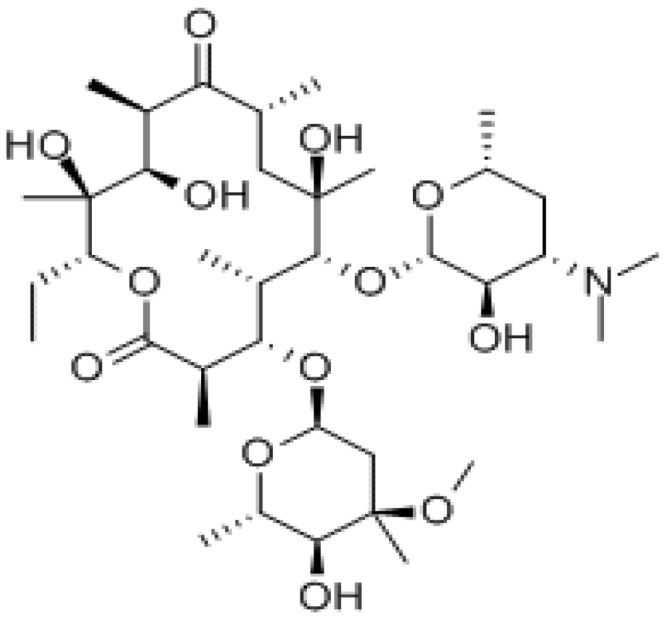
Chemical structure of the erythromycin A molecule.

Erythromycin is a mixture of macrolide antibiotics produced by a strain of *Streptomyces erythreus*, Erythromycin A [EM, (3*R*,4*S*,5*S*,6*R*,7*R*,9*R*,11*R*,12*R*,13*S*,14*R*)-4-[(2,6-dideoxy-3-C-methyl-3-*O*-methyl-α-l-ribo-hexopyranosyl)oxy]-14-ethyl-7,12,13-trihydroxy-3,5,7,9,11,13-hexamethyl-6-[(3,4,6-trideoxy-3-dimethyloamino-β-d-xylo-hexopyranosyl)oxy]oxacyclotetradecane-2,10-dione, Figure 1], which is slightly soluble in water, is the main component [1]. EM binds irreversibly to a site on the 50S subunit of the bacterial ribosome, thus inhibiting the translocation steps of protein synthesis and it may also interfere at other steps, such as transpeptidation. EM is used in the treatment of acne, skin, eye and respiratory tract infections caused by susceptible strains of microorganisms [2]. 

**Figure 2:**
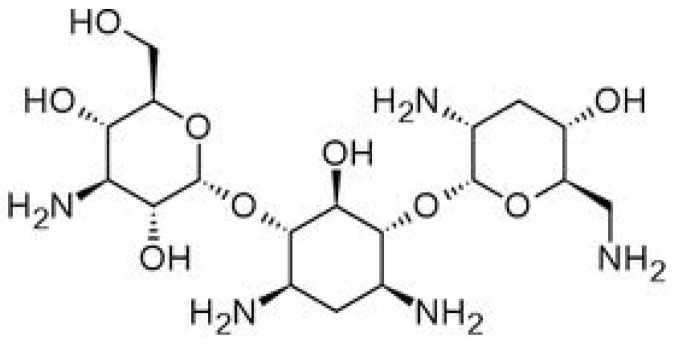
Chemical structure of the tobramycin molecule.

Tobramycin [TOB, 4-*O*-(3-amino-3-deoxy-α-d-glucopyranosyl)-2-deoxy-6-*O*-(2,6-diamino-2,3,6-trideoxy-α-d-ribohexopyranosyl)-l-streptamine, Figure 2] is an aminoglycoside antibiotic freely soluble in water [3]. TOB is effective against a wide variety of Gram-negative bacteria, especially the *Pseudomonas* species and some Gram-positive pathogens. It acts by inhibiting synthesis of protein in bacterial cells and is used in combination with other antibiotics in the treatment of infected post-operative wounds, diabetic foot or other soft-tissue bacterial diseases, gynecologic, ocular and respiratory infections, osteomyelitis, bacteremia and sepsis [4,5].

Dendrimers are relatively new class of compounds which are expected to play crucial and promising role in the biomedical field. They are defined as highly branched, globular, synthetic polymers with well defined size and molecular weight. The structures of dendrimers consist of a central core, layers of branched repeated units which are radically attached to the central core and functional end groups [6,7]. The architecture and unique properties of these macromolecules make them a good platform for drug delivery. Drug molecules can be either encapsulated in empty interior cavities of dendrimers or attached to their multivalent surfaces through covalent conjugation or electrostatic interaction [6,8,9]. Recently, many researchers focused on dendrimers as potential antimicrobial compounds [10,11,12] or agents improving antifungal or antibacterial activity of existing chemotherapeutics [13,14]. 

Polyamidoamine dendrimers (PAMAM) are water-soluble, nonimmunogenic, biocompatibile compounds [15,16,17] and their highly-branched nature could provide enormous surface area that generate great reactivity to microorganisms. Owning to their unique properties, PAMAM have been also evaluated for improvement of drug solubility and drug permeation and as delivery systems for bioactive substances, including antimicrobial agents [18,19,20,21]. Literature data reveal that higher-generations (G4–G6) of PAMAM-NH_2_ dendrimers show stronger toxicity, which hinders further exploration of these compounds as antibacterial compounds [22]. Simultaneously, size of the dendrimer affects its ability to penetrate the bacterial cell and therefore its antibacterial activity - it was found that PAMAM G3 dendrimers were more effective antibacterial agents than PAMAM G5 and lower generation of carbosilane dendrimers were more effective than higher generations [23,24].

The aim of this work was to investigate the influence of PAMAM-NH_2_ and PAMAM-OH dendrimers generation G2 and G3 on antibacterial activity of antibiotics with different solubility in water: hydrophobic erythromycin and freely soluble tobramycin. Furthermore, water solubility of erythromycin in the presence of PAMAM dendrimers was also examined.

## 2. Results and Discussion

### 2.1. Influence of PAMAM Dendrimers on Antibacterial Activity of Erythromycin and Tobramycin

Bacterial resistance to commonly used antimicrobial drugs remains a major challenge for infection treatment. Moreover, many antimicrobial drugs are difficult to administer because of their low water-solubility, cytotoxicity to healthy tissues or rapid degradation. Their antimicrobial activity against intracellular microbes may be also severely limited by poor permeability. Recent studies revealed the efficacy of the use of dendrimers against infectious diseases. It has been shown that some dendrimers themselves possess antibacterial or antifungal activity [10,11,12]. Furthermore, many chemotherapeutics have been successfully incorporated into dendrimer nanoparticles or attached to their functional groups for improving solubility and therapeutic efficacy [22,23,25,26]. The general mode of antimicrobial action of dendrimers is interaction with lipid bilayers which may cause destabilization of the membrane by creating small holes and in the consequence cell lysis [24,25,27,28]. Moreover, dendrimers are able to favour the interaction of the drug with its target or help with its penetration through membranes [26,29]. Among PAMAM dendrimers harbouring a variety of functional groups, amino-terminated PAMAM possess the strongest antibacterial activity. It is generally assumed that protonated amino groups on PAMAM promote the disruption of anionic bacterial cell membranes through electrostatic interactions and they are necessary for the rupture of the lipids, although other interactions might also occur. It was shown that PAMAM-OH and PAMAM-COOH alter the outer membrane of some bacteria, but the underlying mechanism by which these dendrimers induce changes needs to be investigated [30,31,32].

The results concerning the effect of PAMAM dendrimers on the *in vitro* antibacterial activity of EM and TOB obtained by broth dilution method are shown in Table 1, Table 2, Table 3, Table 4. Both of tested antibiotics are chemically weak bases with quite different water solubility (EM is hydrophobic, TOB is hydrophilic). The obtained data of microbiological assay indicated that the presence of PAMAM dendrimers had no influence on Minimum Inhibitory Concentration (MIC) value of EM (Table 1). Although, for strains of *Staphylococcus aureus*, a slight decrease (2- and 4-fold) in value of MBC in the presence of PAMAM-NH_2_ G2, G3 and PAMAM-OH G3 were observed (Table 2). As pure PAMAM dendrimers displayed antibacterial activity against tested strains at a much higher concentration (data not shown), the enhanced bactericidal activity should not be contributed to dendrimers itself. Interestingly, there was no influence of PAMAM on antibacterial potency of TOB and even slight decrease of its activity was noted (Table 3 and Table 4). It is suggested that TOB, as a hydrophilic compound, could be probably complexed to PAMAM molecules and not available for an antibacterial effect.

Moreover, inconsiderable effect of PAMAM dendrimers on the antibacterial activity of tested compounds might be due to the specific construction of the bacterial cell wall. The cell wall of Gram positive (+) bacteria contains a thick layer (*i.e.*, 20–50 nm) of cross-linked peptidoglycan (PG), which may restrict penetration or impair the interaction between the cell membrane and the surface groups of PAMAM dendrimers. Gram negative (−) bacteria cell walls comprise a thin PG layer (~10 nm) but contain an additional outer membrane making the bacteria even more resistant to an external attack. This structure confers resistance to hydrophobic compounds including detergents and contains as a unique component, lipopolysaccharides, which increase the negative charge of cell membranes and are essential for structural integrity and viability of this bacteria [27,28,33,34], therefore molecules of PAMAM were probably unable to effectively interact with this bilayer and cause its destabilization.

**Table 1:** Minimum Inhibitory Concentration-MIC (in µg/mL) of erythromycin (EM) in the presence of PAMAM-NH_2_ and PAMAM-OH dendrimers generation 2 (G2) and generation 3 (G3).

Name of the strain	MIC (µg/mL)				
	EM	EM + PAMAM-NH_2_		EM + PAMAM-OH	
		G2	G3	G2	G3
*Staphylococcus aureus* ATCC 29213 G(+)	0.5	0.5	0.5	0.5	0.5
*Enterococcus faecalis* ATCC 29212 G(+)	1	1	1	1	1
*Staphylococcus aureus* (clinical strain) G(+)	0.5	0.5	0.5	0.5	0.5
*Enterococcus faecalis* (clinical strain) G(+)	>512	>512	>512	>512	>512
*Escherichia coli* ATCC 25922* G(−)	−	−	−	−	−
*Pseudomonas aeruginosa* ATCC 27853* G(−)	−	−	−	−	−
*Acinetobacter baumannii* LMG 1025* G(−)	−	−	−	−	−
*Klebsiella pneumonia* ATCC 700603* G(−)	−	−	−	−	−
*Enterobacter cloacae* ATCC 700323* G(−)	−	−	−	−	−

* strains not susceptible to EM.

**Table 2:** Minimum Bactericidal Concentration - MBC (in µg/mL) of erythromycin (EM) in the presence of PAMAM-NH_2_ and PAMAM-OH dendrimers generation 2 (G2) and generation 3 (G3).

Name of the strain	MBC (µg/mL)					
	EM	EM + PAMAM-NH_2_			EM + PAMAM-OH	
		G2	G3		G2	G3
*Staphylococcus aureus* ATCC 29213 G(+)	16	8	8		16	8
*Enterococcus faecalis* ATCC 29212 G(+)	8	8	8		8	8
*Staphylococcus aureus* (clinical strain) G(+)	16	4	4		16	8
*Enterococcus faecalis* (clinical strain) G(+)	>512	>512	>512		>512	>512
*Escherichia coli* ATCC 25922* G(−)	−	−	−	−	−	−
*Pseudomonas aeruginosa* ATCC 27853* G(−)	−	−	−	−	−	−
*Acinetobacter baumannii* LMG 1025* G(−)	−	−	−	−	−	−
*Klebsiella pneumonia* ATCC 700603* G(−)	−	−	−	−	−	−
*Enterobacter cloacae* ATCC 700323* G(−)	−	−	−	−	−	−

* strains not susceptible to EM.

**Table 3:** Minimum Inhibitory Concentration - MIC (in µg/mL) of tobramycin (TOB) in the presence of PAMAM-NH_2_ and PAMAM-OH dendrimers generation 2 (G2) and generation 3 (G3).

Name of the strain	MIC (µg/mL)				
	TOB	TOB + PAMAM-NH_2_		TOB + PAMAM-OH	
		G2	G3	G2	G3
*Staphylococcus aureus* ATCC 29213 G(+)	1	1	1	1	2
*Enterococcus faecalis* ATCC 29212 G(+)	16	32	32	16	16
*Staphylococcus aureus* (clinical strain) G(+)	1	1	1	1	1
*Enterococcus faecalis* (clinical strain) G(+)	>512	>512	>512	>512	>512
*Escherichia coli* ATCC 25922 G(−)	1	1	1	2	2
*Pseudomonas aeruginosa* ATCC 27853 G(−)	0.25	0.5	0.25	0.25	0.25
*Acinetobacter baumannii* LMG 1025 G(−)	0.125	0.125	0.125	0.125	0.125
*Klebsiella pneumonia* ATCC 700603 G(−)	4	4	4	4	4
*Enterobacter cloacae* ATCC 700323 G(−)	2	2	2	2	2

**Table 4:** Minimum Bactericidal Concentration - MBC (in µg/mL) of tobramycin (TOB) in the presence of PAMAM-NH_2_ and PAMAM-OH dendrimers generation 2 (G2) and generation 3 (G3).

Name of the strain	MBC (µg/mL)				
	TOB	TOB + PAMAM-NH_2_		TOB + PAMAM-OH	
		G2	G3	G2	G3
*Staphylococcus aureus* ATCC 29213 G(+)	4	4	4	4	4
*Enterococcus faecalis* ATCC 29212 G(+)	16	64	64	16	16
*Staphylococcus aureus* (clinical strain) G(+)	4	4	4	4	4
*Enterococcus faecalis* (clinical strain) G(+)	>512	>512	>512	>512	>512
*Escherichia coli* ATCC 25922 G(−)	1	1	1	2	2
*Pseudomonas aeruginosa* ATCC 27853 G(−)	0.25	0.5	0.25	0.25	0.25
*Acinetobacter baumannii* LMG 1025 G(−)	0.25	0.25	0.25	0.25	0.25
*Klebsiella pneumonia* ATCC 700603 G(−)	4	4	4	8	4
*Enterobacter cloacae* ATCC 700323 G(−)	2	2	2	2	2

### 2.2. The Influence of PAMAM Dendrimers on Solubility of Erythromycin

**Figure 3:**
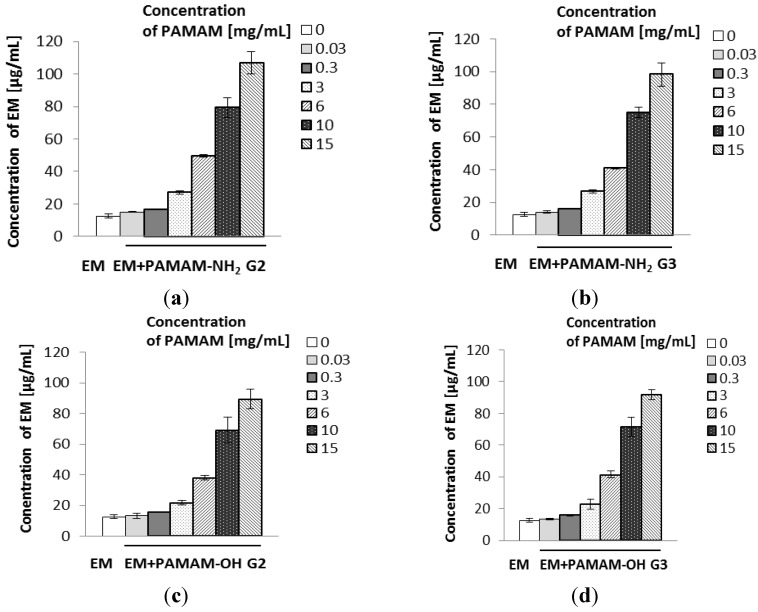
Solubility of erythromycin in the presence of various concentrations of PAMAM-NH_2_ generation 2 (**a**), PAMAM-NH_2_ generation 3 (**b**), PAMAM-OH generation 2 (**c**), PAMAM-OH generation 3 (**d**).

The results of the solubility studies are presented in Figure 3. It was observed that all examined PAMAM dendrimers significantly enhanced the solubility of EM. The solubility of EM in the dendrimers solutions increased in an approximately linear manner with an increase in dendrimer concentration. PAMAM-NH_2_ dendrimers were slightly more effective than PAMAM-OH (PAMAM-NH_2_ G2 dendrimer [15 mg/mL] enhanced the solubility of EM approximately 8.5-fold, from 12.53 µg/mL to 106.93 µg/mL). It could be hypothesized that the solubility enhancement of EM, which is a hydrophobic weak base (pKa = 8.88) [1,2], was rather a consequence of encapsulation and hydrophobic interactions between poorly-soluble drug and internal cavities of PAMAM dendrimers than attachment to their surface groups. Furthermore, there are tertiary amines in internal cavities of PAMAM, which may interact with the atoms of EM molecules by hydrogen bond formation. Interestingly, despite an increase in solubility of EM, there was no considerable effect of PAMAM on its antibacterial activity (Table 1 and Table 2).

## 3. Experimental

### 3.1. Materials

Tobramycin was received as a gift sample from Warszawskie Zaklady Farmaceutyczne (Warsaw, Poland), erythromycin was from Pharma Cosmetics (Krakow, Poland). PAMAM dendrimers generation 2 (G2) and generation 3 (G3) with amine or hydroxyl surface groups, dimethyl sulfoxide (DMSO) and phosphoric acid were purchased from Sigma Aldrich (St. Louis, MO, USA). Acetonitrile HPLC grade was obtained from POCH (Gliwice, Poland). Dipotassium phosphate was from Chempur (Piekary Slaskie, Poland). Cellulose esters (HA) membrane filters 0.45 µM were derived from Millipore (Billerica, MA, USA).

### 3.2. Bacterial Strains

Control strains: *Staphylococcus aureus* ATCC 29213, *Enterococcus faecalis* ATCC 29212, *Escherichia coli* ATCC 25922, *Pseudomonas* *aeruginosa* ATCC 27853, *Klebsiella pneumonia* ATCC 700603, *Enterobacter cloacae* ATCC 700323 were from American Type Culture Collection (Microbiologics^®^, St. Cloud, MN, USA); *Acinetobacter baumannii* LMG 1025 was obtained from BCCM/LMG Bacteria Collection (Belgian Co-ordinated Collections of Microorganisms, Ghent University, Belgium). Clinical strains (*Staphylococcus aureus*, *Enterococcus faecalis*) were isolated from clinical samples obtained from patients treated in departments of the University Hospital in Białystok (Poland). The bacteria were identified by the VITEK 2 GN card and the automatic system VITEK 2 (BioMerieux, Durham, NC, USA) according to manufacturer’s instructions.

### 3.3. Antibacterial Agents

A stock solution of 5,120 µg/mL was prepared by dissolving EM in sterile DMSO, TOB in sterile deionised distilled water and in 10 mg/mL water solutions of PAMAM dendrimers G2 and G3 with amine or hydroxyl surface groups. Series of double diluting solution of above compounds were prepared in sterile Mueller—Hinton broth obtaining the final concentration of EM and TOB in the range from 0.032 µg/mL to 512 µg/mL. Solutions of PAMAM dendrimers and DMSO were also estimated in the absence of tested antibiotics (EM or TOB).

### 3.4. Determination of Minimum Inhibitory Concentration and Minimum Bactericidal Concentration

The Minimum Inhibitory Concentrations (MIC) of tested antibiotics and their combinations with PAMAM dendrimers were determined by the broth microdilution method [29,35]. The medium used for susceptibility testing was Mueller—Hinton broth. The initial density of bacterial strains was 1.5 × 10^8^ colony forming units (CFU) /mL. Inoculums of bacteria (density of 0.5 McFarland scale) were prepared in sterile saline. Then tested strains were suspended in Mueller—Hinton broth to give a final density of 5 × 10^5^ CFU/mL. Solutions of PAMAM, EM, TOB, EM in PAMAM, TOB in PAMAM dendrimers and suspensions of bacterial strains were inoculated onto microtiter plates. The growth control, sterility control and control of antibacterial compounds were used. Plates were incubated under normal atmospheric conditions at 35 °C for 18 h, and afterwards minimum inhibitory concentration (MIC) values have been designated. MIC values were expressed as the lowest concentrations which inhibited growth as judged by lack of turbidity in the tube.

Minimum bactericidal concentration (MBC) was determined by plating 5 µL from the wells showing no visible growth on solid Mueller—Hinton medium and incubating 16–18 h at 35 °C under aerobic conditions. MBCs were defined as the lowest concentration of antimicrobial agent where no bacterial growth on the plates was noted. All experiments were repeated three times. The stock solutions of pure EM or TOB were used as a positive control. Solvent and media controls were used for reference.

### 3.5. Solubility Studies of Erythromycin

The solubility of EM was determined by using shake-flask method and assay was done as follows: an excess amount of antibiotic was added to each vial containing 5 mL of the selected solvents (water or 0.03 mg/mL, 0.3 mg/mL, 3 mg/mL, 6 mg/mL, 10 mg/mL and 15 mg/mL solutions of G2 and G3 PAMAM dendrimers with amine or hydroxyl surface groups). Mixtures were shaken mechanically for 48 h at 25 °C ± 0.5 °C and allowed to stand for 24 h to attain equilibrium. Next, mixtures were centrifuged at 3000 rpm for 15 min, followed by filtration through HA membrane filter (0.45 µM), diluted appropriately with the mobile phase and analyzed by HPLC method at 195 nm against a standard. Mean values from three independent experiments done in duplicate were considered.

### 3.6. HPLC Analysis of Erythromycin

The solubility of EM was determined on an Agilent Technologies 1200 HPLC system equipped with a G1312A binary pump, a G1316A thermostat, a G1379B degasser and a G1315B diode array detector (Agilent, Waldbronn, Germany). Data collection and analysis were performed using Chemstation 6.0 software. Isocratic separation was achieved on a Waters Spherisorb^®^ 5.0 µM ODS 2, 4.6 × 250 mm, 5 μm column (Waters Corporation, Milford, MA, USA). Mobile phase was acetonitrile—phosphate buffer pH 7 (65:35, v/v), the flow rate was 1.0 mL/min and UV detection was carried out at a wavelength of 195 nm [30,31,36,37]. The column was maintained at an ambient temperature. For injection into the HPLC system 20 μL of sample was used. All reagents used for analysis were HPLC grade. The retention time of erythromycin was about 3.0 min. The standard calibration curve was linear over the range of 5–150 μg/mL (R^2^ = 0.9999).

### 3.7. Data Analysis

The results were analyzed by analysis of variance (ANOVA) and multiple comparison were done to check statistical significance. The statistical significance between means was verified by Sheffe’s comparison test accepting *p* < 0.05 as significant.

## 4. Conclusions

The obtained results indicate that PAMAM dendrimers significantly enhance the solubility of poorly water-soluble EM and the increase in EM solubility was mostly dependent on PAMAM concentration. Despite the improvement of solubility, there was no significant effect on enhancement of the antibacterial activity of EM (only slight improvement of EM bactericidal effect for *Staphylococcus aureus* strains was observed). Inconsiderable influence of PAMAM dendrimers on the antimicrobial activity of tested antibiotics might be due to the specific construction of the bacterial cell wall.
